# A novel germline mutation in a patient with nevoid basal cell carcinoma syndrome showing cystic lesion in the lung

**DOI:** 10.1038/hgv.2015.14

**Published:** 2015-06-11

**Authors:** Ryo Miyata, Manabu Kurosawa, Masaaki Sato, Tomoya Kono, Yasutaka Takubo, Shinsaku Okai, Keisuke Yamada, Reiko Shinkura, Hiroshi Date, Fumihiko Matsuda

**Affiliations:** 1 Department of Thoracic surgery, Kyoto University Graduate School of Medicine, Kyoto, Japan; 2 Department of Diagnostic Pathology, Kyoto University Hospital, Kyoto, Japan; 3 Department of Immunology, Faculty of Bioscience, Nagahama Institute of Bio-Science and Technology, Nagahama, Japan; 4 The Center for Genomic Medicine, Kyoto University Graduate School of Medicine, Kyoto, Japan

## Abstract

Nevoid basal cell carcinoma syndrome (NBCCS) manifests multiple defects involving the skin, endocrine and nervous systems, eyes and bones. Mutations in the patched homologue 1 (*PTCH1*) gene are the underlying causes of NBCCS, leading to aberrant cell proliferation through constitutive activation of the hedgehog signaling pathway. We identified a novel frameshift mutation (c.1207dupT) of *PTCH1* in a NBCCS patient, which might explain multiple cystic lesions and neoplastic growth in the patient.

Nevoid basal cell carcinoma syndrome (NBCCS), also called Gorlin–Goltz syndrome, is a rare multisystemic disease inherited in an autosomal-dominant manner. Historically, it was first reported in 1894 for a patient with multiple basocellular carcinomas.^1^ In 1960, Gorlin and Goltz established a classical triad that characterizes this syndrome: multiple basocellular epitheliomas, keratocysts in the jaws and bifid ribs.^2^ Its estimated incidence varies from 1 in 57,000 to 256,000 among various studies, with a male-to-female ratio of 1:1.^3^ The pathogenesis of NBCCS is attributed to abnormalities linked to the long arm of chromosome 9 (q22.3-q31). Various kinds of mutations in the patched homologue 1 (*PTCH1*) gene, a tumor-suppressor gene, are considered as its molecular origin.^4^ *PTCH1* has an important role for embryonic structuring and cell cycle, and thus its genetic alteration can cause a key event for the development of the disease. It is important to genetically diagnose the disease to prevent its fatal consequences such as multiple skin cancers and other malignant tumors.

A 19-year-old female with the first episode of a primary spontaneous pneumothorax was referred to our hospital. Her left lung was completely collapsed in chest X-ray, although she had almost no respiratory symptoms. She was a non-smoker without any history of pulmonary diseases. She was born at her parents ages in early thirties, and there is no other cases of NBCCS in her family. She had undergone surgery for four times because of keratocystic odontogenic tumor (formerly called odontogenic keratocyst) in the upper jaw. Despite chest tube drainage for 5 days, air leakage persisted and thoracoscopic lung resection was selected. Intraoperatively we found pleural adhesion at the superial segment of the left lower lobe. The visceral pleura of the left lung appeared generally edematous. A cystic lesion was found at the lowest part of the dorsobasal segment of the left lower lobe and was resected with a stapler. Interestingly, the surface of the lesion appeared unusually thick for a bulla with an appearance similar to other parts of the visceral pleura (Figure 1a). Macroscopically, the cystic lesion had smooth surface and unevenly thickened wall (Figure 1b,c). There remained persisted air leakage for 7 days postoperatively and eventually she underwent rethoracotomy; since then there has been no evidence of relapse to date for more than 20 months. We diagnosed her as NBCCS because she fulfilled two major criteria for NBCCS: bilamellar calcification of the falx cerebri and keratocystic odontogenic tumor (Supplementary Table 1).^5^

Sections of 4 μm in thickness were used for hematoxylin and eosin staining and immunocytochemical analysis (Figure 2). For immunostaining, antibodies were used to detect alpha-smooth muscle actin and desmin (Dako corporation, Code No. N1584 and N1526, respectively). The detection was on the basis of the Labeled Streptavidin Biotin method (Bench Mark GX VENTANA).

The study was approved by a local ethical committee and informed consent was obtained from the patient and parents. Genomic DNA was extracted from peripheral blood leukocytes with a standard method using proteinaseK and phenol. Nine pairs of oligonucleotide primers were designed for the amplification of the 23 exons of the *PTCH1* gene (Supplementary Table 2). PCR was performed using PrimeSTAR Max DNA Polymerase (Takara Bio, Shiga, Japan) according to the manufacturer’s protocol. PCR products were resolved on a 1% agarose gel and purified with the Wizard SV Gel and PCR Clean-Up System (Promega, Tokyo, Japan). Sequencing of PCR products was performed on an ABI 373A automated sequencer (Life Technologies). The sequences obtained were aligned with a genomic sequence (NG_007664) as well as a full-length complementary DNA sequence of *PTCH1* (NM_000264.3). To confirm the results of direct sequencing, PCR products with P3F and P3R covering exon 4 to exon 8 were cloned into pGEM-T Easy vector (Promega) and sequenced with T7 and SP6 vector primers. The exon numbers and the annotation of the detected mutation were given on the basis of the mRNA sequence of NM_000264.3 according to the recommendation of the Human Genome Variation Society (http://www.hgvs.org/mutnomen/refseq.html).

The histological examination of the cystic lesion revealed the followings. The cysts were lined by columnar cells with no atypia (Figure 2a), and underlying stroma was mainly composed of loose connective tissue with inflammatory cells (Figure 2b) and the dense bundles of bland spindle cells (Figure 2c). They were confirmed to be smooth muscle cells using immunohistochemistry (Figure 2d,e). These structures resembled respiratory tract tissue, and the cyst was covered with fibrous thickening visceral pleura (Figure 2f). These findings were not the histological feature in the pneumothorax; therefore, hamartoma-like lesion or reactive change possibly associated with NBCCS should be considered.

We have undertaken a genetic analysis of the *PTCH1* gene using direct sequencing. Two genetic alterations were identified in *PTCH1* of the patient. The first was located in exon 8 and was an insertion of T nucleotide (c.1207dupT) in one of the two alleles, leading to a premature truncation of the PTCH1 protein with 33 aberrant amino acids in its C terminus (Figure 3a). This mutation was localized in the first extracellular loop of the PTCH1 protein carrying 12 transmembrane domains. The insertion was confirmed by sequencing each allele using the cloned PCR products. We also examined whether this insertion was observed in her parents’ genome. As the mutation was not present in either of her parents, it was a *de novo* germ-line mutation specific to the patient (Figure 3a). The second genetic alteration, a T to C transition that leads to an amino-acid substitution from leucine to proline, was found in exon 23 for one of the two alleles of the patient (Figure 3b). The genetic variant was registered in dbSNP as rs357564, and the frequency of C allele was reported as 0.384 in the Japanese population of the International HapMap Project. It was indeed observed in her unaffected mother. We therefore concluded that c.1207dupT was the causative genetic mutation.

NBCCS manifests multiple defects involving the skin, nervous system, eyes, endocrine system and bones. Because different disease phenotypes appear in multiple organs of patients at their early ages, an early and accurate diagnosis is extremely important for clinicians to evaluate signs and symptoms of the disease and to undertake appropriate treatments. The diagnostic criteria for NBCCS were established^6^ and modified in 1997.^5^ According to the criteria, the diagnosis can be established when the patient satisfies either two major or one major and two minor clinical manifestations (Supplementary Table 1). In our case, two major manifestations, namely, keratocystic odontogenic tumor and bilamellar calcification of falx cerebri (Figure 1d and e), and two minor manifestations of macrocephaly adjusted for height and frontal bossing were observed. Although there are some other clinical manifestations related to the disease as shown in Supplementary Table 1, there are no such complications so far in the patient. To our knowledge, there was only one case of a NBCCS patient with large congenital cyst reported in a family consisting of four NBCCS cases.^7^ The patient showed similar clinical manifestations with our case: the onset of pneumothorax in their juvenile and the presence of smooth muscle bundles in the cyst wall. Although no genetic analysis was undertaken for this family, the four patients were most likely to share the same genetic mutation. The reason for such a cyst in only one of the four cases was speculated as either an additional manifestation of NBCCS or simply a coincidental finding in one case.

Sequencing analysis of the whole exons of *PTCH1* identified a novel single-nucleotide insertion in c.1207dupT resulting in a frameshift at the amino acid. Most of the abnormal mRNAs were reported to be degraded via a mechanism called nonsense-mediated mRNA decay. In the present case, however, a truncated PTCH1 protein with 33 aberrant amino acids at its carboxyl terminus is likely to be synthesized and caused disease phenotypes. The PTCH protein has 12 transmembrane domains and two large extracellular loops.^8^ PTCH1 is involved in the hedgehog (HH) pathway and served as a receptor for the HH ligand.^9^ Two extracellular loops are coded in exons 2–9 and exons 15–18 in *PTCH1*,^10^ and the second extracellular loop of PTCH1 works as receptor for the HH ligand.^11^ HH signaling is a key regulator of embryonic development and tumorigenesis controlling cellular proliferation. Therefore, truncated PTCH1 protein because of this insertion in exon 8 could result in an aberrant cell cycle progression and neoplastic growth.^12^

## Figures and Tables

**Figure 1:**
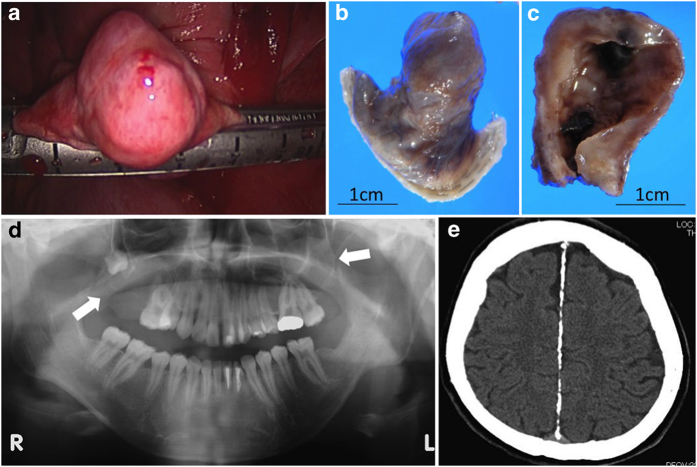
Intraoperative finding of the pleura and cystic lesion (**a**). Gross appearance of the resected cystic lesion with smooth surface (**b**) and the cut section with unevenly thickened cyst wall (**c**). An orthopantomogram showing cystic lesions in the mandibular region (white arrows) (**d**). Brain-computed tomography showing intracranial ectopic calcifications of the falx cerebri (**e**). L, left; R, right.

**Figure 2:**
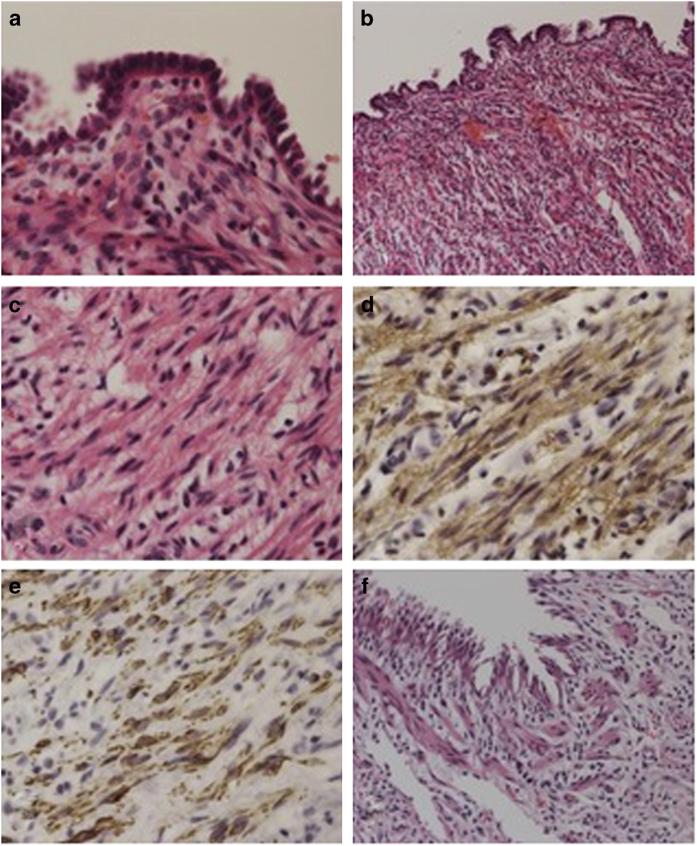
Histological appearance of the resected cystic lesion. The cyst lumen was covered by columnar epithelium (**a**). The underlying stroma was mainly composed of loose connective tissue with inflammatory cells (**b**). Many bundles of bland spindle cells are identified (**c**). The spindle cells are positive for alpha-smooth muscle actin (**d**) and desmin (**e**). These bundles surrounded the cyst lumen, which resembles respiratory tract tissue (**f**).

**Figure 3:**
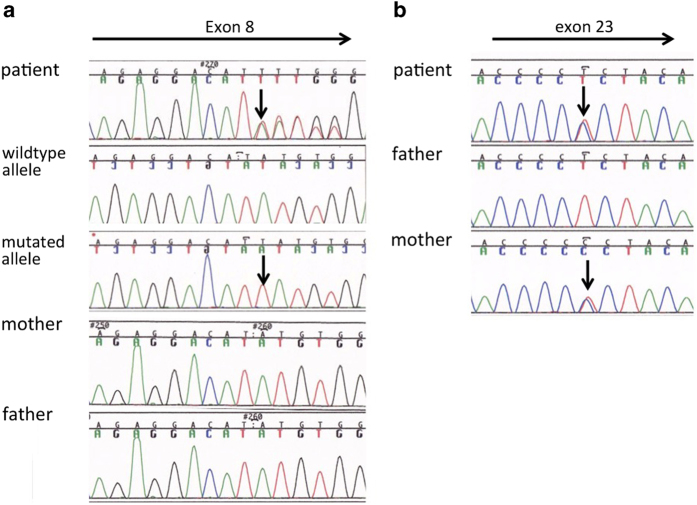
Sequencing analysis of the *PTCH1* gene. (**a**) Direct sequencing results using PCR products covering exon 8 of the *PTCH1* gene in the patient and parents. An insertion of a T nucleotide c.1207dupT causing a frameshift is shown by an arrow. Allele-specific sequencing results of cloned PCR products of the patient with a vector primer are also shown. As cloned PCR fragments were sequenced with reverse primer relative to gene orientation, electropherograms were inverted to match their direction with the other results. (**b**) Direct sequencing results of a T to C transition identified in exon 23 (indicated by an arrow).
