# Supplementary material for: A novel germline mutation in a patient with nevoid basal cell carcinoma syndrome showing cystic lesion in the lung
Source: Hum Genome Var. 2015 Jun 11;2:15014–. doi: 10.1038/hgv.2015.14 (PMC4785575; doi:10.1038/hgv.2015.14)
Supplement: Supplementary Table 1 [file hgv201514-s1.doc]

**Supplementary table 1.** Diagnostic criteria for Gorlin-Goltz syndrome

| Major clinical manifestations | |
| --- | --- |
| 1) | Multiple (>2) BCCs or one under 20 years |
| 2) | Keratocystic odontogenic tumor of the jaws proven by histopathology |
| 3) | Three or more palmar pits (3 or more) |
| 4) | Bilamellar calcification of the falx cerebri |
| 5) | Bifid, fused, or splayed ribs |
| 6) | First-degree relative with NBCCS |
| Minor clinical manifestations | |
| 1) | Macrocephaly determined after adjustment for height |
| 2) | Congenital malformation: cleft lip or palate, frontal bossing, "coarse face", moderate of severe hypertelorism |
| 3) | Other skeletal abnormalities: sprengel deformity, marked pectus deformity, marked syndactyly of the digits |
| 4) | Radiological abnormalities: bridging of the sella turcica, vertebral anomalies such as hemivertebrae, fusion or elongation of the vertebral bodies, modeling defects of the hands and feet, or flame-shaped lucencies of the hands or feet |
| 5) | Ovarian fibroma |
| 6) | Medulloblastoma |
