# Supplementary material for: A novel germline mutation in a patient with nevoid basal cell carcinoma syndrome showing cystic lesion in the lung
Source: Hum Genome Var. 2015 Jun 11;2:15014–. doi: 10.1038/hgv.2015.14 (PMC4785575; doi:10.1038/hgv.2015.14)
Supplement: Supplementary Table 2 [file hgv201514-s2.doc]

**Supplementary table 2.** Primer information

| Exons | Primers for amplification | Additional primers for sequencing |
| --- | --- | --- |
| 1-2 | P1F: TCTTCCGCGAACTGGATGTG |  |
|  | P1R: TGCGCTGGCGAATATCTCTA |  |
|  |  |  |
| 3 | P2F: CAGGTAGTCAGATAACAGAT |  |
|  | P2R: TGGACAAATGCTCTAATTAG |  |
|  |  |  |
| 4-8 | P3F: TATCATTTCGAATTTGCACT | EX7F:AAGGAGCATTTGTTTTGATG |
|  | P3R: TGGTGAAAATGAAGAATTGC | EX9F:GGTACATCAATCTATTAAAT |
|  |  |  |
| 9-12 | P4F: CTGTCGAGGCTTGTGGAAGT | EX12F:GTGCTGGTGGCAGAGTCCTA |
|  | P4R: ACCGCAGACATGGGATGCTG |  |
|  |  |  |
| 13-15 | P5F: TAAAAGCATCTTTATTTTCG | EX15F:CTGTGTAAAATGGGTATTC |
|  | P5R: AATCTAACGCTCTCATAATC |  |
|  |  |  |
| 16-18 | P6F: ACCCCGCTAGGACCAGGGTC | EX18F:AAGCTCAGCTTCTGTGCTCTC |
|  | P6R: GTAGAATAAACATATTACGG |  |
|  |  |  |
| 19-20 | P7F: TCATTGTTTTGATCTGAACC |  |
|  | P7R: TCCTTGACCTTCTGATCCAC |  |
|  |  |  |
| 21-23 | P8F: CACACGGAGGGTGGCGCGAT | EX23F: GAAGGGGAGGTTAATACGCA |
|  | P8R: GATGCCGAGAACCCCAGGAG |  |

Referred sequences were NG_007664 for genomic DNA and NM_000264.3 for mRNA.

The sequence of primers is shown in the 5' - 3' order.
